# Bioremediation of contaminated soil and groundwater by *in situ* biostimulation

**DOI:** 10.3389/fmicb.2023.1258148

**Published:** 2023-11-06

**Authors:** Martin Romantschuk, Katariina Lahti-Leikas, Merja Kontro, Polina Galitskaya, Harri Talvenmäki, Suvi Simpanen, John A. Allen, Aki Sinkkonen

**Affiliations:** ^1^Faculty of Biological and Environmental Sciences, University of Helsinki, Lahti, Finland; ^2^Research Institute for Environmental Studies, Parede, Portugal; ^3^Natural Resources Institute Finland (Luke), Horticulture Technologies, Turku, Finland

**Keywords:** biostimulation, bioremediation, biodegradation, *in situ*, bacteria, organic contaminants

## Abstract

Bioremediation by *in situ* biostimulation is an attractive alternative to excavation of contaminated soil. Many *in situ* remediation methods have been tested with some success; however, due to highly variable results in realistic field conditions, they have not been implemented as widely as they might deserve. To ensure success, methods should be validated under site-analogous conditions before full scale use, which requires expertise and local knowledge by the implementers. The focus here is on indigenous microbial degraders and evaluation of their performance. Identifying and removing biodegradation bottlenecks for degradation of organic pollutants is essential. Limiting factors commonly include: lack of oxygen or alternative electron acceptors, low temperature, and lack of essential nutrients. Additional factors: the bioavailability of the contaminating compound, pH, distribution of the contaminant, and soil structure and moisture, and in some cases, lack of degradation potential which may be amended with bioaugmentation. Methods to remove these bottlenecks are discussed. Implementers should also be prepared to combine methods or use them in sequence. Chemical/physical means may be used to enhance biostimulation. The review also suggests tools for assessing sustainability, life cycle assessment, and risk assessment. To help entrepreneurs, decision makers, and methods developers in the future, we suggest founding a database for otherwise seldom reported unsuccessful interventions, as well as the potential for artificial intelligence (AI) to assist in site evaluation and decision-making.

## Introduction

Contaminated soils have become a persistent environmental problem that has not received sufficient attention when compared with polluted waters and air. This is illustrated by the fact that there has been a water directive at the EU-level since the year 2000 (EUR-Lex, 2000) and an air quality directive since 2008 (EUR-Lex, 2008), but only now the framework and concrete steps toward protection, restoration and sustainable use of soils are set by the EU in EU soil strategy for 2030 (European Commission, 2017, 2023). As part of this new strategy, a new Soil Monitoring Law has been proposed to address key soil threats in the EU, such as contamination (European Commission, 2017, 2023). The new law puts in place a solid and coherent monitoring framework, makes sustainable soil management the norm in the EU and requests EU Member States to identify potentially contaminated sites, investigate these sites and address unacceptable risks for human health and the environment (European Commission, 2017, 2023). Along with risks to the environment, polluted soil and environmental contaminants pose both direct and indirect risks to human health and well-being (Parajuli et al., 2018; Roslund et al., 2019). The soil, especially clay soil, forms a reservoir for pollutants that can desorb into water and volatilize, or desorb and evaporate into the air (Pukkila and Kontro, 2014; Mattsson et al., 2015).

Bioremediation and *in situ* treatment methods as concepts usually generate initial enthusiasm and favorable comments from regulatory authorities and remediation contractors. The actual implementation of these techniques in practice is, however, often met with skepticism. Authorities commonly do not want to take responsibility for the effectiveness of what are seen as novel techniques, and entrepreneurs want to use old, familiar methods and avoid unnecessary financial risks (Hannu Silvennoinen, personal communication).

Exceptions to these skeptical attitudes can be found in both the public and private sectors. Hannu Silvennoinen, CEO of Nordic Envicon, referencing Dahl et al. (2013), has pointed out that “even reaching a partial goal of decreasing the volume [of contaminated soil] that requires excavation saves a substantial amount of both [in terms of] money and environment[al impact].” Nikunen et al. (2017), in calculating the results from the SOILI program, found that *in situ* treatment could achieve average savings of 30% over traditional methods.

The use of monitored natural attenuation may also be a viable solution whenever a thoroughly performed site analysis and risk assessment (RA) has concluded that there is no risk for spread of the contaminant and that contaminant concentration reduction takes place naturally at a satisfactory rate (Simpanen, 2016; Fernandez-Lopez et al., 2022). RA is a sensible way to evaluate whether a site no longer poses an environmental or health risk. Locally applied regulations and tools may be needed, but the principle has backing both in the literature (Öberg and Bergbäck, 2005; Latawiec et al., 2010) and in regulatory recommendations (Ministry of the Environment, 2014). Even so, realistic bioremediation plans are often abandoned due to unrealistic and counterproductive demands to reach below target concentrations in every sample. It is therefore commendable that authorities in Finland (FINLEX, 2007), based on international and EU trends, recommend RA rather than absolute preset targets.

If an organic contaminant is principally biodegradable, but shows no trend of reduction in concentration, there is likely to be one or more bottlenecks for active degradation that need to be removed. Identifying these bottlenecks is therefore crucial. Furthermore, all bottlenecks need to be identified and amended in order to generate a desired rate of biodegradation and contaminant removal (Table 1).

**TABLE 1:** Microbial remediation bottlenecks and suggested solutions (activities elucidated in the text).

– Temperature not optimal for degradation (in most cases too low):
ᵒ Injection of steam, hot air or hot water
ᵒ Electric heating
ᵒ Electrokinetic pumping raises the temperature slowly
– Lack of oxygen or alternative electron acceptors:
ᵒ O_2_, NO_3_^–^, SO_4_^2–^, Fe^3+^, CO_2_ to stimulate oxidation
– Lack of carbon source or electron donor
ᵒ Addition of lactate or other carbon source to stimulate reductive dechlorination, degradation of N or P-rich contaminants, etc.
– Lack of key nutrients
ᵒ Addition of N (ammonia, nitrate, urea, and methylene urea) in case of, e.g., oil hydrocarbons, etc. C-rich substrates
ᵒ Addition of P if concentration too low – phosphate buffer also used for pH adjustment
ᵒ Addition of micronutrient mixture
– Microbes with suitable degradation capacity not present or not induced:
ᵒ Bioaugmentation with microbes carrying genes/pathways missing in indigenous microbes
ᵒ Co-metabolism activated by addition of simple degradation-pathway inducing substrates
ᵒ Microbes redistributed by electrokinetic pumping of other liquid moving actions
– Poor bioavailability
ᵒ Addition of biosurfactants (e.g., cyclodextrin and rhamnolipid)
ᵒ Bioaugmentation with surfactant-producing microbes

To speed up bioremediation, the local situation should be modified to allow sufficiently vigorous microbial growth and metabolism in a manner where the target contaminant or some component of it is made to be the limiting factor for growth. If this is achieved, other microbes competing for resources (e.g., electron acceptors) cannot out-compete the relevant degraders. This should be kept in mind, for example, when adding carbon and energy sources, such as easily degradable biomass during bioaugmentation, since competing metabolism may deplete the oxygen supply. If the projected degradation takes place in aerobic conditions any addition that can be oxidized may cause delays in target contaminant degradation (Simpanen et al., 2016b; Talvenmäki et al., 2021), while in contrast, anoxic reducing conditions may be desirable if de-chlorination or denitrification is the goal (Conrad et al., 2010).

In some situations co-degradation of contaminants upon, e.g., addition of general stimulation of microbial activity has been suggested (Conrad et al., 2010) but this approach may lead to degradation of the stimulating agent rather than the contaminant (Talvenmäki et al., 2021).

**FIGURE 1:**
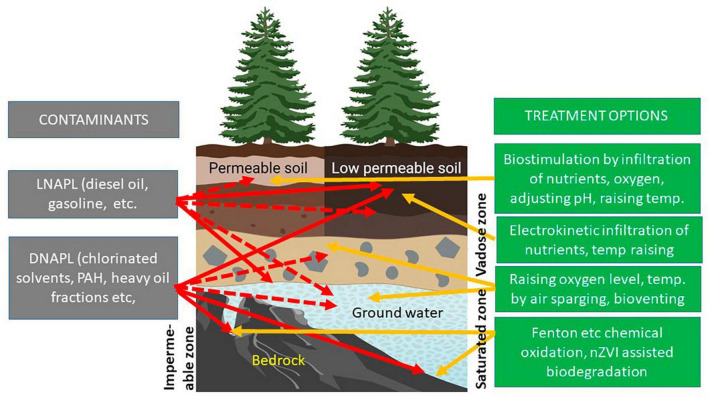
Contaminated scenarios and options for activity. Assumed situation: contamination is not fresh and rapid changes no longer seen. Based on analysis of soil, ground water, and local conditions, a strategy is drawn up, and if possible, tested in laboratory conditions. Red arrows: contaminant location – solid, main contaminant locality; dashed, residual concentrations or area of limited spread. Yellow arrows: suggestion of method and target for intervention. Ground water may contain plumes, depending on contaminant solubility.

Environmental parameters such as temperature, availability of oxygen and nutrients, should be optimized and targeted for the bacterial consortium with the highest degradation potential. Thus, if the contaminant is a petroleum hydrocarbon (PHC), this contaminant should be made the limiting carbon and energy source, while if the contaminant is an organonitrogen or organophosphate pesticide, nitrogen or phosphorus, respectively, should be made the limiting factor (Figure 1).

The target values to reach with regards to temperature, redox potential, pH, etc. have to be chosen based on an educated guess. Soil temperatures at 1–2 m below the surface varies only a little with the seasons, and deeper down it is almost constant. In boreal conditions subsurface soil temperatures are below 10°C and the soil does not freeze, partly due to the insulating snow cover. At a site in South-Eastern Finland the ground temperature stayed at 4–7°C during a 4 month period, September to December at a depth of 1.5 m (Suni et al., 2007). In such conditions the microbial profile is predominantly psychrophilic and mesophilic, and excessive temperature increases appear to limit microbial activity (Suni et al., 2007), while a modest *in situ* elevation to between 15 and 20°C has been observed to boost microbial activity (Simpanen et al., 2018; Talvenmäki, 2020).

## Classification of contaminants, remediation, and site evaluation methods

Soil organic pollutants can be classified into the following, partly overlapping groups: explosives, pesticides (DDT and its metabolites, triazines, etc.), halogenated hydrocarbons (polychlorinated biphenyls, PCBs), flame-retardants (polybrominated diphenyl ethers, PBDEs), surfactants, PHCs and related plastic pollution, polyaromatic hydrocarbons (PAHs), dioxins, and medical waste/pharmaceuticals (Jones and de Voogt, 1999; McGrath et al., 2017; Kraemer et al., 2019; Verla et al., 2019; Tauqeer et al., 2021). Environmental remediation of these pollutants usually starts with identifying the problem, i.e., the extent of the pollution; contaminants, their concentrations and stability of hazardous compounds; soil type(s) including physical, chemical, and microbiological properties of the matrix; water flow conditions; etc. Additionally, the effect of removing the contamination source on the lifetime of the contaminate should be estimated based on its degradation rate (Pande et al., 2020; Patel et al., 2020; Bala et al., 2022). A computational framework is under development for predictive bioremediation to assess environmental fate in remediation approaches based on molecular dynamics simulation, and biodegradation pathway predictions to overcome limited information about the site studies (Singh et al., 2021). Remediation performance must be monitored and that may require the installation of inspection system components, such as piping, pumps, and valves. Parameters typically monitored include pH, dissolved oxygen, temperature, mineral nutrient levels, flow rates (air and water), and pumping rates. Microbial community composition, including the number of specific contaminant-degrading microbial subgroups, and the overall microbial population density should be determined. The decision to end remediation activities should be based on the effectiveness of the remediation results (Pande et al., 2020; Patel et al., 2020; Bala et al., 2022).

In recent years a number of novel methods for remediation of contaminated sites have been developed. Description and classification of these methods and of contaminated sites are presented in the scientific literature and in guidelines (Romantschuk et al., 2000; Volchko et al., 2014; Rosén et al., 2015; Singh et al., 2015; Norrman et al., 2020; Table 1).

Remediation technologies can be subdivided into two broad classes, *ex situ* and *in situ*. *Ex situ* techniques include the more “traditional” remediation methods, which typically require the excavation and transportation of contaminated soils for treatment. *In situ* techniques, in contrast, do not include excavation, transportation and isolated treatment of the contaminated sites. While *ex situ* methods, such as landfarming and biopiles, may incorporate bioremediation in the treatment process, as a rule, *in situ* techniques are gentler, less invasive, and thus have a less negative impact on the environment (Nikunen et al., 2017; USEPA, 2017; Bajagain et al., 2020a; Fernandez-Lopez et al., 2022). Various *in situ* methods offer important advantages, especially when traditional, off-site “dig and dump” approaches are ruled out by technical or economic demands. When a successful outcome can be achieved *in situ*, the demands of sustainability are achieved on all fronts: the reduced need for transportation means reduced CO_2_ emissions and fuel use, resulting also in significant financial savings (Simpanen, 2016). Social and/or economic benefits are reaped from the fact that normal activities at the site can continue and buildings and structures are undisturbed during the treatment, as opposed to off-site methods which are often invasive and disruptive (Simpanen et al., 2016b).

In natural attenuation, or passive *in situ* treatment, the contaminated soil is remediated by naturally occurring biological, chemical, and physical processes. With bioavailable and easily degradable pollutants a successful end result may be achieved with natural attenuation alone (Penttinen, 2001) largely based on redox-reactions (Tuomi and Vaajasaari, 2004). However, some monitoring is still needed for natural attenuation to be considered an actual treatment method (Wilms et al., 2023).

Active *in situ* methods for removal of organics from soil may include biological as well as physical or chemical methods. In this review we focus on biodegradation but touch upon physico-chemical techniques when combined or necessary to achieve biodegradation via biostimulation (Table 2).

**TABLE 2:** Description of various *in situ* soil remediation methods including some of their benefits and disadvantages (general references: Romantschuk et al., 2000; Penttinen, 2001; Nathanail et al., 2007; USEPA, 2007, 2013; Tomei and Daugulis, 2013).

*In situ* methods	Principle	Benefits/Disadvantages	References (specific)
**Biological**			
Monitored natural attenuation	Contaminant reduction by natural (biological, physical, and chemical) processes, efficacy of soil cleanup and risk reduction are monitored	+ Cost-effective − Long-term process − Suitable only for some contaminants	Mulligan and Yong, 2004; Rügner et al., 2006.
Biostimulation	creating a favorable environment for microbial contaminant degradation, includes the addition of nutrients, oxygen, electron acceptor/donor, and controlling the temperature and pH	+ Cost-effective − Suitable only for degradable contaminants − Low contaminant bioavailability reduces degradation efficiency	Suni et al., 2007; Kauppi et al., 2011; Tyagi et al., 2011
Bioaugmentation	Addition of enriched/acclimated microbes having a degradation capacity to specific contaminant	− Possibly ineffective due to the failure adaptation of microbes to environmental conditions	Sarand et al., 2000; Gao et al., 2022
Bioventing (biosparging)	Injection of air/oxygen above (or below) the water table using low pressure and venting rate optimized to maximize biodegradation	+ Minimal site disturbance − Unsuitable in low-permeable or heterogeneous soils	Thomé et al., 2014
Nanobioremediation	NMs facilitate bioremediation by enhancing the growth of microorganisms, immobilizing the remediating agents or through induced production of remediating microbial enzymes	+ Faster than bioremediation alone + Cost-effective + Suitable for various contaminants − Spreading of NMs to reach the contaminants is challenging − NMs may have toxic effects on organisms	Kumari and Singh, 2016; Bhatt et al., 2022
Chemical*			
Chemical oxidation/reduction	Injection of oxidizing or reducing agents into soil to cause a complete or partial degradation of contaminant	+ rapid degradation also in high contaminant concentrations − Non-selective degradation can cause negative environmental impacts	Talvenmäki et al., 2021; Zheng et al., 2022
Physical*			
Soil vapor extraction (Lim et al., 2016)	Applying a high vacuum to the soil to induce air flow and removal of volatile contaminants May improve aerobic conditions	+ Cost-effective, well-known technique − Low efficiency in wet and dense soil	Lim et al., 2016; Simpanen, 2016
Electro-remediation	Inducing an electric field into the soil to cause movement and desorption of charged contaminants May improve spread of nutrients and degrading microbes May improve bioavailability	± Most efficient in fine grained, wet soils + Suitable also for inorganic contaminants	Suni et al., 2007; Huang et al., 2012

*Listed for cases that can be used in combination with or as pre- or post-treatment for bioremediation.

## Microbial bioremediation – forms, prerequisites, and conditions

Bioremediation is a process that mainly relies on the use of microorganisms to remove pollutants from contaminated areas in order to regain the original condition of the site. The process involves biological degradation of organic pollutants to carbon dioxide and water, or to less toxic intermediates under natural conditions (Romantschuk et al., 2000). Although plants and their rhizosphere can also positively influence conditions, the focus here is on active bioremediation via biostimulation.

In bioattenuation, biostimulation, and bioaugmentation, microorganisms, either indigenous or introduced, play the main role (Kauppi et al., 2011, 2012; Simpanen et al., 2016a,b; Talvenmäki et al., 2021). The choice of a particular bioremediation technology should be based on careful planning and is dependent on several factors, such as the characteristics of the polluted soil or water, state of the indigenous microbial population and the type and concentration of pollutants (Nikunen et al., 2017).

In the case of readily biodegradable organic compounds such as PHC, mineralization of the contaminant is largely dictated by bacteria (Romantschuk et al., 2000; Mukherjee et al., 2015; Simpanen et al., 2016a), but recent research shows that Archaea may also be involved (Yan et al., 2018, 2020). To facilitate bioremediation in such cases, soil conditions need to be optimized for bacterial activity and proliferation, which will accelerate removal of the pollutant. Bottlenecks for bacterial activity, such as lack of oxygen or an alternative electron acceptor, suboptimal nutrient balance, and moisture level and/or temperature should be targeted for optimization efforts (Margesin, 2000; Simpanen et al., 2016a,b; Cavazzoli et al., 2022, 2023). Suitable pH and improved bioavailability of the contaminant can also be obtained via additives (Penttinen, 2001; Peltola et al., 2006; Suni et al., 2007; Talvenmäki et al., 2021). An aquatic carrier is often used with an obvious biostimulative role in itself (Khalladi et al., 2009). Aerobic degradation is often more efficient than anaerobic (Tarasov et al., 2004; Kauppi et al., 2011), encouraging the addition of oxygen as a gas straight into the soil or the aquatic phase (Penttinen, 2001), or as a breakdown product of chemicals such as peroxide, persulfate, ozone or permanganate (Tarasov et al., 2004; Goi et al., 2006; Yen et al., 2011; Nykänen et al., 2012; Simpanen et al., 2016a; Bajagain et al., 2018, 2020b). The dissolvability of oxygen is comparatively low; alternatives like nitrate and sulfate sidestep this problem but come with a heightened risk of groundwater pollution (Chen et al., 2022).

In bioremediation, nitrogen and phosphorus are the pivotal macronutrients (Chaîneau et al., 2005) whereas the former is often the sole limiting nutrient in terrestrial systems (Chapin et al., 2011). The unbalanced C_org_:N:P ratio resulting from the excess carbon of the organic contaminant can thus be optimized with additions of nitrogen. The optimal C_org_:N ratio of 10:1 is difficult to achieve with urea, the most commonly used additive, since the ammonium carbonate producing reaction of urea hydrolysis often increases soil pH to undesired levels (Peltola et al., 2006) that in turn escalates the hydrolysis reaction, resulting in nitrogen leaching trough volatilization of ammonium (Jones et al., 2007). Ideally the availability of nitrogen should be adjusted to the bioavailable portion of the carbon rich contaminant rather than the total amount. This can be facilitated by using methylene urea (MU), a condensation product of urea and formaldehyde in which the length and degree of polymerization affects the pace of release (Koivunen et al., 2004; Peltola et al., 2006). The MU is degraded at a rate that portions out nitrogen in balance with the available carbon. The benefits of using nitrate as the nitrogen source are that it can also function as an electron acceptor (Kim et al., 2013; Chen et al., 2022) and is readily soluble in water as opposed to gaseous oxygen.

## Biostimulation and reductive dechlorination

The emerging organohalide contaminants are widely occurring in the environment and suitable for bioremediation by reductive dechlorination or halorespiration. These include chlorinated solvents, PCBs with many different molecular structures, and promising remediation results have also been obtained with perfluoroalkyl and polyfluoroalkyl substances, as well as halogenated flame retardants (Huang and Jaffé, 2019; Yu et al., 2020; He et al., 2021). Contaminants such as chlorinated solvents and PCBs in their oxidized states are not susceptible to oxidation under aerobic conditions, leading to microbial biodegradation via reductive dechlorination under anaerobic conditions, which is the main pathway for their degradation (Dutta et al., 2022). In the reductive dechlorination, cellular energy is produced by replacing one or more chlorine atoms with an electron-donating hydrogen molecule made available by adding organic substances (Tiehm and Schmidt, 2011).

One of the most commonly cited examples of reductive dechlorination is the metabolism of chloroethenes, i.e., the dechlorination of tetrachloroethene (PCE) to trichloroethene (TCE), then to dichloroethene isomers (*cis*-DCE and *trans*-DCE), and finally to vinyl chloride (VC) and ethane. Among these metabolites, PCE and TCE, as the most oxidized molecules, are most susceptible to reductive dechlorination, while VC has slower reaction rates and risk of accumulation, co-metabolism being one option for VC degradation. Chloroethenes with the highest chlorine number inhibit the reductive dechlorination of molecules with less chlorine (Tiehm and Schmidt, 2011). Reductive dechlorination of PCBs is more complex and slower than that of chloroethenes, and its progression is related to geochemistry and the specific microorganisms present in the polluted environment (Wiegel and Wu, 2000; Xiao et al., 2020). Brominated molecules, such as PBDEs, and decabromodiphenyl ether can also be degraded by microbial reductive debromination (Gerecke et al., 2005; Robrock et al., 2008). Although the C-F bond is one of the strongest in nature, *Acidimicrobium* sp. oxidizing ammonium to nitrite and reducing Fe(III) was capable of defluorinating per- and polyfluoroalkyl substances using hydrogen as the sole electron donor instead of ammonium (Huang and Jaffé, 2019).

Reductive dehalogenation can be performed by iron- and sulfate-reducing microbes, as well as by methanogenic and fermentative microbial communities, i.e., reductive dechlorination is a cometabolic or respiratory process (Löffler et al., 1999; Yuan et al., 2021). The energy released in reductive dechlorination of 135–187 kJ/mol is much greater than the energy available from sulfate reduction (38 kJ/mol) and methanogenesis (31 kJ/mol), while as much as 228 kJ/mol of energy is released in Fe(III) reduction (Löffler et al., 1999; Dolfing, 2000). Microorganisms that respire chloroethenes and PCBs most typically belong to *Firmicutes*, *Chloroflexi*, or *Proteobacteria*. In particular, the genus *Dehalococcoides* is related to the reductive dechlorination of chlorinated hydrocarbons. Other possible genera for dechlorination include *Anaeromyxobacter*, *Dehalobacter*, *Dehalogenimonas*, *Desulfitobacterium*, *Desulfoluna*, *Desulfomonile*, *Desulfovibrio*, *Desulfuromonas*, *Geobacter*, *Propionibacterium*, and *Sulfurospirillum* (Xiao et al., 2020). *Acidimicrobium* sp. strain A6 has been reported to defluorinate per- and polyfluoroalkyl substances (Huang and Jaffé, 2019). In addition, methane oxidizing microorganisms, such as *Methylomonas* sp. and *Methylosinus* sp. have been associated with dechlorination through co-metabolism (Xiao et al., 2020). Typically, a decomposing microbial community develops naturally in an environment exposed to pollutants, and the same microorganisms live in similar environments in nature. Because of this, remediating microbes should be isolated from an environment similar to where they are intended to be used in bioaugmentation (Liu et al., 2015).

Slow-growing, anaerobic *Dehalococcoides* spp. with a preference for neutral pH, carry as many as 14 different reductive dechlorinase genes in their genome. The genes of different strains are typically non-identical, and therefore *Dehalococcoides* strains have variable ability to degrade organohalides (Hölscher et al., 2004). The dechlorination pathways may even differ between two *Dehalococcoides* strains (Cheng and He, 2009). The strain *Dehalococcoides ethenogens* is among the rare isolates that can completely dechlorinate PCE and metabolites to ethene. Examples of different reductive dechlorinase genes include: the *tceA* gene, which encodes a TCE reductive dehalogenase enzyme that catalyzes the dechlorination of TCE, 1,2-dichloroethane, 1,2-dibromoethane, and dichloroethenes to ethene (Magnuson et al., 2000); the enzyme VC-reductive halogenase of *Dehalococcoides* sp. strain VS, encoded by the *vcrA* gene, which reduces VC and all dichloroethene isomers to ethene (Müller et al., 2004); and the *bvcA* gene of *Dehalococcoides* sp. strain BAV1, which is involved in the reductive dechlorination of VC to ethene (Krajmalnik-Brown et al., 2004). Real-time PCR assays have been designed to detect *Dehalococcoides* from 16S rDNA and genes *bvcA*, *vcrA*, and *tceA* to monitor the performance of the reductive dechlorination remediation process, and evaluate solutions for problems in remediation practices (He et al., 2003; Johnson et al., 2005; Ritalahti et al., 2006; Krzmarzick et al., 2012).

In a review of remediation practices at 191 chlorinated solvent-contaminated sites in the United States and Europe, monitored natural attenuation was implemented at approximately 30% of sites, while additional treatment approaches were applied at other sites (McGuire et al., 2004). With PCBs, natural, *in situ* reductive dechlorination proceeds slowly (Wiegel and Wu, 2000; Xiao et al., 2020). Sources of suitable nutrients and electron donors such as phosphorus or nitrogen, and organic substrates are usually insufficient to achieve natural attenuation. To overcome these problems, biostimulation with nutrient delivery can be used to stimulate the native dechlorinating microbial community, and this has been applied in many aquifers *in situ* to enhance degradation of chlorinated solvents. The substrates used to provide electrons for reductive dechlorination include methane, acetate, pyruvate, methanol, ethanol, lactate, propionate, butyrate, glucose, etc. Fermentation of these carbon sources produces electron donors, such as hydrogen. In addition to acting as an electron donors, added carbon sources can generally promote microbial growth, oxygen consumption, and creation of anoxic conditions required for reductive dechlorination. Lactate, one of the most commonly used substrates, and ethanol both results in the fermentation of acetate (Hood et al., 2008; Blázquez-Pallí et al., 2019). As the remediation of chlorinated hydrocarbons is often a long-duration process, slowly releasing carbon and electron sources can also be used, such as whey, fatty acid mixtures, vegetable oil, carboxymethylcellulose, polylactic acid, polyhydroxyalkanoates, etc. (Borden et al., 2007; Lee et al., 2008; Kocur et al., 2016; Pierro et al., 2017; Sun et al., 2022).

Other methods combined with biostimulation of reductive dechlorination include the addition of micro- and nano-scale zero valent iron (ZVI), which enhances the simultaneous abiotic dissipation of contaminants (Xie et al., 2017).

## Biosurfactants as support

Methods based on water circulation tend to work well for hydrophilic compounds, but the bioavailability of hydrophobic compounds presents a challenge (Khalladi et al., 2009; Martins et al., 2009; Banat et al., 2021), especially since it is closely connected to the risk of contaminant mobilization. Biostimulation in itself does not appear to mobilize contaminants (Simpanen et al., 2016a,b). In a simulation of an oil transportation accident, biostimulation was compared to natural attenuation and chemical oxidation. During the monitoring period, biostimulation had an insignificant effect on the oil contents in the soil, but a pronounced effect on the oil flushed downwards with the water. The logic behind the observation is that microbes do not readily degrade oil that is bound to soil particles, but whatever is dissolved from the particles is bioavailable and degraded (Simpanen et al., 2016a). The bioavailability of hydrophobic compounds can be enhanced by the use of surfactants and biosurfactants that lower the effort required to overcome surface tension by situating themselves at the interface of solvents which differ in polarity (Del Valle, 2004; Khalladi et al., 2009; Talvenmäki et al., 2021; Eras-Muñoz et al., 2022). The use of surfactants accelerates the dissolution of PHCs such as diesel oil from the soil, and may result in the formation of biodegradable aqueous forms, desorption, and even emulsification of hydrocarbons (Calvo et al., 2009; Ayed et al., 2015; Bajagain et al., 2020b). Biosurfactants significantly vary in their structure and molecular weight. Their common characteristics is a high surface activity since they consist of hydrophobic (fatty alcohols, fatty acids, and unsaturated and saturated hydrocarbons) and hydrophilic (oligo-, mono- or polysaccharides, proteins, and peptides) moieties (Lin, 1996). Properly functioning biosurfactants are supposed to improve degradability by dissolving and emulsifying hydrophobic contaminants such as oil compounds or PAHs, while remaining inert long enough so as not to cause competition for resources. If, however, the surfactant itself is too easily degradable it may cause vigorous microbial activity that consumes the oxygen, nitrogen, etc., leaving the contaminant untouched (Talvenmäki et al., 2021). The rate of emulsification should also be matched by the rate of degradation, otherwise there is a risk of mobilizing the contaminant into the soil and groundwater (Simpanen et al., 2016b). A similar risk arises if a component in the contaminant spectrum is dissolved but non-degradable.

Many hydrocarbon degrading microbial species are able to produce biosurfactants that can be both intra- and extracellular. Among them, bacteria belonging to *Pseudomonas, Acinetobacter, Bacillus, Rhodococcus*, and *Stenotrophomonas* genera as well as fungi belonging to *Candida, Torulopsis*, and other yeast genera producing glycolipids (e.g., rhamnolipids, sophorolipids, and trehalose lipids), lipopeptides (e.g., iturin, surfactin, fengycin, lichenysin, and viscosin), and other minor biosurfactant classes, are the most studied (Karlapudi et al., 2018). For *in situ* bioremediation in cold climates, it is meaningful that many microbially produced surfactants have been shown to be stable at low temperatures and under extreme salinity and pH conditions (Baek et al., 2007; Sharma et al., 2022). Field studies of biosurfactant efficacy in the bioremediation of oil contaminated soils are limited, in contrast to lab studies. However, they demonstrate a significant increase in hydrocarbon decomposition rates under various environmental conditions (Tumeo et al., 1994; Nikolova and Gutierrez, 2021). High bioremediation rates were achieved when biosurfactants were used to enhance biostimulation or bioaugmentation (Baek et al., 2007; Cameotra and Singh, 2008; Kang et al., 2010; Tahseen et al., 2016). Bioaugmentation with the bacteria applied in a surfactant foam was shown to be efficient even at very low temperatures (Jeong et al., 2015).

Particularly interesting from an *in situ* point of view is the potential to harness biosurfactant producing bacterial strains for colonization of contaminated soils. *Pseudomonas aeruginosa* producing rhamnolipid was tested for crude oil degradation in combination with nutrition optimization, with encouraging results (Ma et al., 2016) and attempts have been made to implement this concept in *in situ* scenarios. Monitoring the efficacy is, however, challenging (Decesaro et al., 2017).

Besides crude oil and oily sludge, biosurfactants were demonstrated to enhance bioremediation of PAHs, pesticides and other hydrophobic organic pollutants. The underlying mechanism for organic compounds coincides with that for crude oil, in that an improvement of wettability and emulsification of the pollutant results in higher biodegradability (Raj et al., 2021).

## Biostimulation vs. bioaugmentation

Biostimulation requires the presence of microbes possessing the proper degradative capabilities. If the contamination is old pollution with reduced bioavailability, it is highly likely that a degradative community is present, and the slow natural attenuation is due to suboptimal conditions slowing microbial activity (Suni et al., 2007; Kauppi et al., 2011; Dehnavi and Ebrahimipour, 2022). Bioaugmentation may be a viable alternative if the contaminant is highly recalcitrant and/or fresh. However, even freshly contaminated sites can be assumed to harbor microbes capable of degradation of principally biodegradable substances, such as mineral oil (Simpanen et al., 2016a; Banet et al., 2021) or PAHs from creosote (Koivula et al., 2004). Bioaugmentation was also found to significantly improve the remediation results compared to biostimulation alone for soil that lacked indigenous oil degraders (Chaudhary et al., 2021). Examples of the contrary are, however, also reported even in newly contaminated soil. Pilot scale tests in pristine, sandy soil with a humus soil top layer showered with a controlled amount of gasoline-diesel mix showed that biostimulation resulted in cleaning of leachate oil fractions, while natural attenuation did not (Simpanen et al., 2016a). The tested approach was adapted for a real truck accident site with good results (Tervo, 2013; Tervo and Kolehmainen, 2016).

Bioaugmentation is the addition of desired microbes to a system in order to accelerate remediation. This may be beneficial in situations where the indigenous microbial community is suspected to have decreased significantly. For example, in combination with chemical oxidation, such as using Fenton’s reagent for oily hotspots, an inoculum may speed up recovery of microbial degradation, since the high concentration of hydrogen peroxide more or less disinfects the soil locally, even beyond the zone of effective treatment (Talvenmäki et al., 2021). Thermal treatment of hotspots may have similar effects on adjacent areas where the thermal degradation as such does not reach, but the applied heat is still enough to kill soil microflora.

Bioaugmentation is not often needed (Suni et al., 2007; Kauppi et al., 2011) and may even worsen the situation as compared to the application of biostimulation alone. Bioaugmentation only works if conditions are suitable for the microbes to function, and so should always be complemented with biostimulation to guarantee appropriate conditions for microbial activity. In many cases bioaugmentation has no influence on microbial community composition. Even with sufficient biostimulation the added microbes may not perform well *in situ*, and soon disappear (Sarand et al., 2000; Kauppi et al., 2011). If degradation is limited by electron acceptor availability, adding another source of easily degradable biomass (the added bacterial cells) may slow the degradation of relatively recalcitrant compounds by consuming the available oxygen (Talvenmäki et al., 2021).

Checking the presence of key degradative genes has been used to predict the likelihood of successful remediation of soils contaminated with specific organic compounds (Baelum et al., 2010; Sinkkonen et al., 2014; Castro-Gutierrez et al., 2022; Wilms et al., 2023). A prerequisite to determine the suitability of this approach is that the degradation pathway is known, at least in part (Salminen et al., 2008). This was accomplished for atrazine (Sagarkar et al., 2013; Nousiainen et al., 2014) and chlorophenols in mesocosms (Sinkkonen et al., 2014) and in field applications (Mikkonen et al., 2018) where changes in gene frequencies using qPCR analysis correlated to degradation activity.

Naturally, the presence of microbes carrying relevant genes does not guarantee success, but the absence of such genes can be a strong indicator of poor chances even if biostimulation is applied. In such cases bioaugmentation may have an initial effect on degradation even if the introduced microbial strain or strains do not necessarily survive (Sarand et al., 2000). If the genes encoding for the introduced strain’s degradation capacity are located in a mobilizable position, such as in conjugative plasmids or transposons (Sipilä et al., 2010; Jeffries et al., 2018) this capacity can spread to the indigenous population as observed for the TOL-plasmid (Sarand et al., 2000) in the rhizospheres of pine.

Degradation pathways that have been introduced via a carrier strain may also evolve rapidly under selective pressure within the original carrier or after transfer to new recipients if their original genes are not exactly suited for the task. In this way degradation potential can rapidly diversify toward additional methylated phenols in humus soil (Sarand et al., 2001). This exploits the plasticity of the genes and gene clusters when functioning in natural environments (Ray et al., 2016).

When bioaugmentation is used, it may be of interest to monitor the survival and performance of the introduced microbes. Detecting the presence of the bioaugments can be done with certainty if a detectable marker gene or other unique genetic element is inserted in connection to the operon or gene cluster of interest (Sarand et al., 2000; Boureau et al., 2002), but this may require too much case-specific preparation to be widely applicable.

A simpler approach is to design primer pairs targeting genes of interest. Such targets can be genes known to be active in degradation of the contaminant of interest (Yan et al., 2018) but this will not distinguish between indigenous and introduced microbes. By combining PCR based identification of specific target genes and, e.g., 16S rRNA genes (Juhanson et al., 2009) introduced strains can be recognized in field experiments even years after application. Target gene presence can be quantified with qPCR, which, in combination with analysis of the diversity of 16S rRNA genes in the soil, may give a good estimate of the survival of the introduced strain(s) (Shahi et al., 2016; Li et al., 2021, 2023).

An interesting and novel method that, in addition to presence also monitors activity, is the use of metabolomics to get a snapshot of the *in situ* metabolic state of a dechlorination-active microbiome following bioaugmentation (May et al., 2022). This technology could be adapted for any genetically well-characterized situation, but performance monitoring requires a high level of technical capability.

An example of exploitation of native degradation capacity was seen when the degradation of pyrene was monitored in humus soil. Humus contains complex, heteropolymeric macromolecules, such as lignin and humic substances, which microbes are capable of degrading. Based on this observation, the conclusion was drawn that microbes normally exposed to such complex, natural substances containing a variety of hetero-aromatic compounds, could also adapt to rapidly degrade polyaromates such as pyrene (Koivula et al., 2004). The adaptability of humic microbial populations was also demonstrated regarding chloro-aromates (Kauppi et al., 2012; Sinkkonen et al., 2013). A different, more “organic” form of bioaugmentation is thus to rely on the microbial community of particular environments, that either contain natural substances structurally similar to the contaminant or that have been previously exposed to and cleaned from substances similar to the target compound, and to use transplantation from such environments into the contaminated site. In the case of PAHs (Koivula et al., 2004), herbicides such as triazine (Sinkkonen et al., 2013) and diesel or light fuel oil (Kauppi et al., 2012; Talvenmäki et al., 2021) transplantation of soil harboring microbes with degradative capacity promotes contaminant degradation when compared to pristine control soil of similar origin. With regards to bioaugmentation *in situ*, the challenge that remains in field conditions is the introduction of the microbial population into the contaminated site, but in some situations infiltration has proved to be a good strategy (Tyagi et al., 2011; Talvenmäki et al., 2021). Furthermore, infiltration can be increased by addition of surfactants on remedial agents (Gautam et al., 2019).

Phytoremediation, utilizing plants and their rhizosphere to introduce stimulatory compounds and elements (e.g., C) to contaminated soils, and can be considered an indirect form of biostimulation (Salam et al., 2022 and referenced therein, Yrjälä et al., 2010). However, this large topic is not within the scope of this review.

## The challenge of non-aqueous phase liquids

Hydrocarbons that are poorly soluble in water may spread in soil and travel rapidly as a non-aqueous phase liquid (NAPL) down to the water table (Caetano et al., 2017). Light NAPL, such as diesel or light fuel oil, spreads on the surface of the groundwater at a rate that is influenced by groundwater stream velocity, soil density, and the viscosity of the NAPL at the current local temperature. However, the distribution of the contaminate can be determined by novel methods (Tsai et al., 2020), possibly aiding in targeted removal by pumping or excavation. As long as the contaminant remains a NAPL (i.e., not emulsified or dissolved by the addition of surfactants), degradation is extremely slow, since microbes have access to the compound only at the NAPL/water interface.

Dense NAPLs, such as creosote that contain large proportions of polycyclic aromatic hydrocarbons, but also many synthetic halogenated organic compounds (Tetrachloroethylene, etc.), do not stay on the surface of groundwater, but travel through the water ending up in bedrock fractures in relatively cold conditions (Yeo et al., 2003; Schaefer et al., 2009). Even if the solubility of the compound is low, the contaminant may spoil groundwater reservoirs and drinking water sources. Using bioremediation for such cases is questionable since removing biodegradation bottlenecks such as increasing the temperature, air sparging to induce aerobic conditions, or injection of surfactants is likely to mobilize the contaminant. Such mobilization, on the other hand, increases the NAPL surface area and the NAPL/water interface, thereby increasing the bioavailability of the contaminant, which may be a prerequisite for successful bioremediation. Thus, if all environmental and health RAs point to an acceptably low risk, *in situ* bioremediation may be an option (Baldwin et al., 2017; Schaefer et al., 2017). A viable alternative may be to introduce or attempt to favor microbes that produce biosurfactants *in situ*. Such bacterial preparations are commercially available and have been used with some success (Mariaamalraj et al., 2016).

Before reaching the water table (LNAPL) or impermeable soil layers (DNAPL) a large portion of the NAPL that travels through soil in the vadose zone is adsorbed to soil particles. Factors influencing the proportion of this adsorption include soil structure, soil moisture (Mercer and Cohen, 1990) and organic matter content, as well as properties of the liquid itself, such as viscosity and hydrophobicity. Biostimulation under such conditions requires mobilization of the contaminant to make it bioavailable (Simpanen et al., 2016a). Even when active mobilization is not attempted, biostimulation appears to be active only on the water accommodated fraction (WAF) portion of the contaminant, that is, only the solubilized contaminant portion is degraded (Mercer and Cohen, 1990; Simpanen et al., 2016a). In case the NAPL fraction does not reach the groundwater level, degradation of any WAF may, however, be sufficient to protect the groundwater from being contaminated. In such a situation biostimulation can be used to protect the groundwater (Simpanen, 2016).

## Semi-active forms of *in situ* bioremediation

Different versions of pump-and-treat (Mikkonen et al., 2018; Zha et al., 2019), that act slowly but keep the contaminant plume from spreading, may be considered less risky than active biostimulation or bioaugmentation, and therefore easier to permit. Also chemical treatment such as the Fenton reaction or use of nano-iron (nZVI) has been used. The reaction is faster than with biostimulation, but the risks of mobilizing part of the contaminant may remain high.

Improved efficiency of *in situ* chemical oxidation, used as a pretreatment for bioremediation, was achieved by applying a persulfate-oxidation foam mixed with a surfactant to increase infiltration into the soil (Bajagain et al., 2018). Likewise, surfactant foam containing potassium permanganate has been shown to significantly improved the effectiveness of subsequent biodegradation of PHC (Bajagain et al., 2020b).

Construction of reactive barriers to limit the spread of contaminants in groundwater from an identified source is a relatively low risk action since nothing is necessarily done to the source itself (Liu et al., 2018; Naeimi et al., 2021). The remediating action, however, is slow and can be considered more of a prophylactic measure to protect downstream groundwater (Azubuike et al., 2016) than an active treatment of contaminants from an identified source.

## Nanobioremediation

To tackle the limitations of bioremediation, such as long duration and formation of toxic degradation products, the combinations of nanotechnology and bioremediation (nanobioremediation) has gained significant attention (Grieger et al., 2015; Shahid et al., 2022). Nanotechnology is based on nano-sciences, i.e., the study of structures and materials on a nanoscale (with a dimensions of <100 nm, nanomaterials (NMs)) and the manipulation and application of these materials. In remediation, NMs have certain advantages that larger particles lack. For example, iron particles in nanosize are 10–100 times more reactive than macro-iron particles thanks to the larger surface-area-to-volume ratio (Zhang et al., 1998). Reactivity of the NMs is based on (a) reductive reactions in which NMs work as an electron donor to transform or convert the contaminant to a less toxic and/or less mobile form, and (b) stabilization and sorption in which the NMs work as a sorbent, precipitant, and/or co-precipitant of the contaminant (Cundy et al., 2008).

NMs used in nanobioremediation can be divided according to their production material into inorganic, such as metals, bimetals, metal oxides, ceramics and synthetic polymers, and organic, such as carbon nanotubes, fullerenes, cellulose nanoparticles, and natural polymers (Rizwan et al., 2014). Successful implementation of nanobioremediation is based on the sequenced use of NMs along with biotechnologies. In such cases, NMs are first applied to reduce high concentrations of contaminants to levels favorable for microbial remediation or as an additional source of growth-limiting substrates (Bhatt et al., 2022). As a result NMs may cause the soil microbial community composition to shift in favor of more resistant and faster-degrading microbial groups (Bhatt et al., 2022). For example, zero-valent iron nanoparticles are known to provide an additional source of electron donors, H_2_, to bacteria capable of dehalogenation of chlorinated organics (Bruton et al., 2015) and carbon nanotubes have been found to enhance the growth of some potential contaminant degrading genera (Shrestha et al., 2013). Additionally, microbes and enzymes can be immobilized with NMs to enhance the microbial activity and longevity, to ease the reuse and recovery of microbial cells and allow the protection of cells from adverse environmental conditions (Kumari and Singh, 2016).

Nanobioremediation has been tested successfully for many types of contaminants, such as heavy metals (Kumar et al., 2023), hydrocarbons (Galdames et al., 2020; Chauhan et al., 2023), nutrients (Gibert et al., 2022), chlorinated ethenes (Sheu et al., 2016; Summer et al., 2020), and pesticides (Singh and Saxena, 2022; Sunanda and Ghosh Sachan, 2023). However, some concerns have been raised regarding the safety of NMs, since they may have negative impacts on different organisms, such as plants and microbes once released into the environment (Khati et al., 2018; Bhatt et al., 2022). Especially higher concentrations of NMs have shown some toxicity effects (Brayner et al., 2006). Therefore more research is needed regarding suitable concentrations of NMs and their possible risks should be evaluated before larger scale experiments are conducted. Nevertheless, especially in colder climates where microbial degradation is slower (Mohn and Stewart, 2000; Bajaj and Singh, 2015; Miri et al., 2019), use of NMs to speed up and enhance microbial degradation can be beneficial.

## *In situ* methods used in sequence or in combination

As previously noted, raising the rate of soil and groundwater cleaning by biostimulation in practice means removing biodegradation bottlenecks, or to at least substantially lower the threshold for active biodegradation. All bottlenecks hindering relevant microbial activity need to be removed – adding missing nutrients is not sufficient if oxygen is also lacking or vice versa. This means that a comprehensive analysis of the local situation needs to be undertaken to determine the best strategy in each case. This may appear self-evident, but surveys performed at contaminated sites do not always take into account all of the relevant parameters.

Natural attenuation, biostimulation, bioaugmentation, chemical oxidation or reduction, physical treatments, or a combination of different methods are all potentially viable solutions for the *in situ* remediation of contaminated sites. However, when bioremediation through biostimulation is the chosen course, other methods are almost always included. Thus, the last alternative – a combination of methods – is the most common approach in bioremediation.

Excavation and off-site treatment is often the chosen method for remediation, and may be unavoidable in situations where new building activities will commence within weeks or months of the decision. At the other extreme, natural attenuation may be a valid choice if a site is considered stable, in that the contaminant is not likely to spread to surface or ground waters, or to be exposed at the ground surface. Choosing natural attenuation may make sense when optimizing input-output is a primary concern. Ranking the sites should at that point be based on RA, where environmental (environmental RA) and human health (health RA) issues are prioritized rather than the economic value of a site (discussed further in the next section). Priority for active intervention/remediation should be given to sites where the contaminant situation is not stable, putting adjacent soils and/or groundwater at risk. Monitored natural attenuation, on the other hand, can be a valid choice where the situation is stable and where contaminant levels are reduced at a sufficient rate. With this type of ranking a maximum number of high risk sites can be treated while risk-free sites also progress toward complete removal of contamination.

In most cases *in situ* bioremediation is not as fast as, e.g., excavation and relocation or chemical oxidation with peroxide (Fenton reaction). However, in contrast to excavation, *in situ* treatments can take place beneath buildings and roads without the need for demolition. Also, even when such obstacles do not preclude excavation but there is no particular rush, *in situ* treatment can be considered as a both cost effective and more sustainable option. Removal and transportation of large quantities of soil is not needed, and neither is replacing the excavated soil with pristine soil from elsewhere, significantly reducing the overall environmental impact of the remediation project.

It can be stated that the best results can be achieved by combining methods based on case-by-case evaluation in a goal-oriented manner. The risk is that consultants and contractors favor methods that they represent and/or have expertise in, rather than withdrawing from risky cases. Ideally, a consultant or site evaluator with a sufficiently broad palette of expertise can find combinations of methods and, when needed, combinations of contractors that can tackle the project with a concerted effort. Alternatively, the contractor should represent or have expertise in a sufficient array of techniques so as to be capable of implementing optimal combinations by itself. These consultants and contractors should also be required to acknowledge when the methods that they represent are not likely to work. This means that excavation in some cases may be the most environmentally sustainable solution, but it should never be the only option considered.

## Tools to support action: RA, EE, LCA, and AI in the decision making process

Deciding on a particular course of action for a remediation project should be informed by many different factors to help ensure that the strategy with the lowest overall negative impact and maximum effectiveness is chosen. This is often a complex and difficult task, requiring extensive expertise and resources. According to Sorvari and Seppälä (2010), decision support tools (DSTs) can be useful, especially when used with a multi-criteria decision analysis approach, though most existing tools focus on, e.g., site characterization and/or sampling strategy rather than on the evaluation of remediation technologies. DSTs that make use of RA, eco-efficiency (EE), life cycle assessments and, possibly, artificial intelligence (AI) can be used to simplify and improve the process. Here we discuss aspects of DSTs and some software-based tools that are currently available.

Risk assessment examines the human health and environmental impacts of a project and is a widely used method to determine the need for remediation. Mahammedi et al. (2020) examined RA tools used in different countries and that are tailored for specific types of risk and situations. However, the authors found that many of these were lacking in key respects, such as user-friendliness and ability to accurately assess some risks. Additionally, RAs have significant weaknesses that may limit their usefulness in bioremediation projects. For example, RAs rarely consider which remediation methods are to be used or their overall environmental and social impacts (Sorvari and Seppälä, 2010).

Eco-efficiency, based on the ratio of ecological to economic factors involved in a project, is widely used in contaminated land management. While useful in many contexts, Sorvari and Seppälä (2010) found that a lack of established assessment methods and guidelines may be major barriers to the use of EE locally.

Life Cycle Assessments (LCAs) are typically used to evaluate the environmental impacts of various products, processes, and systems throughout their entire life cycle. This process can also be useful in the selection of strategies for bioremediation. By encompassing all aspects of a project, LCA quantifies its environmental impacts from “cradle to grave,” evaluating energy consumption, emissions, resource use, and potential ecological impacts associated with the bioremediation processes being considered. This makes LCA a potentially powerful technique for identifying optimal remediation strategies while helping to minimizing the project’s overall environmental footprint. Although a properly performed LCA is important, alternatives at a specific location should always be assessed based on a number of factors, including those that are not necessarily dealt with in the LCA process. It is especially important to include: direct threats to human health, environmental safety, stability vs. volatility of the contaminant situation, and urgency of continued and/or future use of the site (Romantschuk et al., 2000; Betrie et al., 2013).

Many software-based DSTs exist that can be useful for the evaluation of bioremediation projects, such as Decerns MCDA,^1^ openLCA,^2^ SimaPro,^3^ Sphera (formerly known as GaBi),^4^ and Umberto.^5^ Additional tools are in use locally in EU member states, the USA, and elsewhere. One example is the PIRTU EE calculation tool (SYKE, 2013) developed in Finland for assessment of contaminated soil and groundwater sites based on the calculation of risks, environmental impact, costs, and other impacts. However, proper use of the tool requires expertise for evaluation of the numerical results produced. Sorvari and Seppälä (2010) also developed a DST based on multi-attribute value theory for Finnish contaminated sites. Another tool is the SCORE developed in Sweden for assessing sustainability of remediation of contaminated soil (Rosén et al., 2015). In the USA, BIOPLUME III^6^ is a sophisticated two-dimensional contaminant transport model used to predict natural attenuation of organic contaminants in groundwater under various conditions, which may be modified to include some types of bioremediation (Rifai et al., 2000). Private consultancy companies have also developed their own tools for assessing sustainable remediation, one example of which is SURE by Ramboll (2023).

Recent developments in AI programs such as natural language generation or large language models may also aid in the evaluation and selection of bioremediation strategies for particular sites. Already these programs, such as the web-based ChatGPT,^7^ are attracting attention in a wide range of fields, such as project management, programming and data analysis (Prieto et al., 2023; Surameery and Shakor, 2023; Zhu et al., 2023). While far from perfect (Lo, 2023; Zhu et al., 2023), given accurate information about site characteristics (i.e., setting, geology, hydrology, *in situ* microbial community, etc.) and the contaminant of interest, it is possible for a web-based program such as ChatGPT to give apparently useful recommendations for effective remediation strategies based on the literature in its training dataset. It is possible to image that such a model, trained on datasets particular to environmental remediation, could produce effective remediation plans that improve outcomes.

Furthermore, in order to prevent repeating the mistakes of others, a database for—grossly under-reported—unsuccessful *in situ* bioremediation cases should be established. Such matters have been discussed, e.g., in a report by the National Research Council (U.S.) (1993), and consultancy companies may keep up their own registers, but no widely accessible, updatable database is, to our knowledge, in use. A question that arises is: who should maintain and validate the contents of such a database? The responsible party would need to ensure the integrity of the contents and prevent its miss-use, for example, by using it to generate false negative publicity for a particular method. Ideally, such a database should be maintained by national or international public authorities or by an NGO.

**FIGURE 2:**
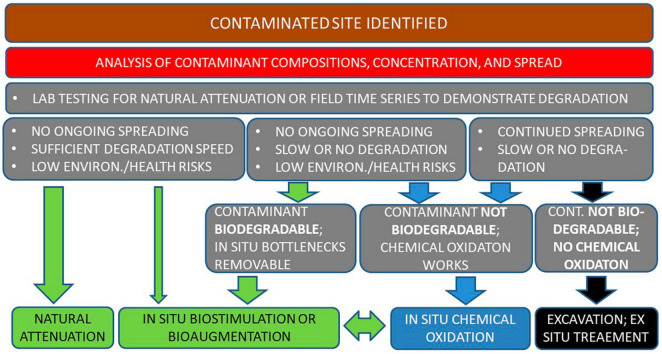
Stepwise progression toward decision for action at a contaminated site. Following site identification (brown box) the contaminant composition, site characteristics and area contaminated are determined (red). A sequence of lab tests (gray) commence to determine degradability, stability, and risk aspects. Depending on lab results field activities are chosen: biological [natural attenuation, biostimulation, and bioaugmentation (green)], chemical oxidation (blue), or excavation/*ex situ* treatment (black) as a last resort. Biological and chemical remediation may in some situations be used in combination or in sequence (vertical double arrow).

Use of these existing and proposed tools can ease the implementation and efficacy of bioremediation, drawing attention to its possibilities, and reducing the temptation to resort to the most common remediation technique: dig and dump. Actions taken to remediate polluted environments created by human activities should be optimized to achieve the maximum effect and improvement from the often limited available resources. Reaching this goal requires thorough planning and pre-testing, based on solid expertise. A proposed order of actions is presented in Figure 2.

## Author contributions

MR: Conceptualization, Writing—original draft, Writing—review and editing. KL-L: Writing—original draft, Writing—review and editing. MK: Writing—review and editing. PG: Conceptualization, Writing—original draft, Writing—review and editing. HT: Writing—original draft, Writing—review and editing. SS: Writing—original draft, Writing—review and editing. JA: Writing—review and editing. AS: Writing—review and editing.

## Footnotes

1
http://decerns.com

2
https://openlca.org

3
https://simapro.com

4
https://sphera.com

5
https://ifu.com/umberto/

6
https://epa.gov/water-research/bioplume-iii

7
https://chat.openai.com
